# Control surface allocation based on offline handling quality simulations for a flying wing aircraft

**DOI:** 10.1007/s13272-025-00906-2

**Published:** 2025-10-22

**Authors:** Salvatore Asaro, Direnc Atmaca, Erik-Jan van Kampen, Roelof Vos

**Affiliations:** Faculty of Aerospace Engineering, Delft University of Technology, Kluyverweg 1, Delft, 2629HS The Netherlands

**Keywords:** Flying wing, Control surface sizing, Offline handling quality simulations

## Abstract

Commercial applications of flying wing aircraft, such as the Flying-V considered herein, can contribute to reducing carbon and nitrogen emissions produced by the aviation sector. However, because of the lack of a tail, all flying wing aircraft have reduced controllability. For this reason, the placement and sizing of the control surfaces along the wing is a nontrivial problem. The paper focuses on solving this problem using offline handling quality simulations based on certification requirements. In different flight conditions, the aircraft must be able to perform a set of maneuvers as defined by the certification specifications. First, offline simulations calculate the minimum control authority required from the elevator, aileron, and rudder to perform each maneuver. Then, based on the global minimum for all maneuvers, the control surfaces are sized and placed along the wings. The aerodynamic model employed uses a combination of Reynolds-averaged Navier–Stokes (RANS) and vortex lattice method (VLM) simulations. The control authority of the control surfaces is estimated with VLM and VLM calibrated with RANS simulations, showing significant differences between the two.

## Introduction

**Fig. 1:**
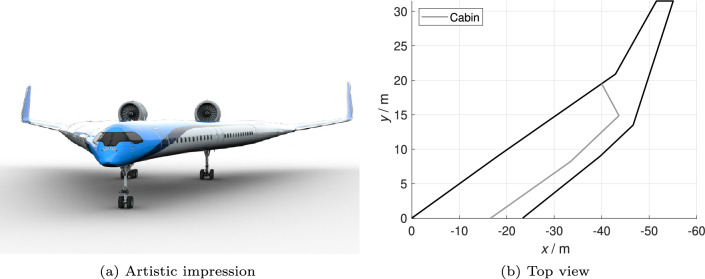
Artistic impression and top view of Flying-V aircraft

The Flightpath 2050 report issued by the Advisory Council for Aeronautics Research in Europe (ACARE) sets an ambitious goal of reducing greenhouse gas emissions [5]. Particularly, a reduction of 75% for CO$_2$ and 90% for NO$_{x}$ is defined as targets with respect to conventional aircraft from 2000s. Novel aircraft designs, such as flying wing aircraft, could reduce drag, contributing to reducing the aviation sector’s footprint. Several flying wing designs are being investigated [4]; most of them feature highly swept inner wings to replace the fuselage, such as the Flying-V [3]. An artist’s impression of the Flying-V is depicted in Fig. 1a and a top view with the cabin layout in Fig. 1b. The airplane has an inner wing with a leading-edge sweep angle of 64$^\circ$ and an outer wing with a leading-edge sweep angle of 39$^\circ$. The wing span is $b= 65$ m, and the mean aerodynamic chord $\overline{c}= 18$ m. The current design, without engines, landing gear fairing, or roughness, shows a maximum lift-to-drag ratio of 24.2 in cruise conditions, with angle of attack $\alpha$ = 3.6$^\circ$ and freestream Mach number $M_\infty$ = 0.85 [11].

The initial design of the airplane features an elevon along the trailing edge of the outer wing that acts as both elevator and aileron for pitch and roll control, respectively. The yaw motion is controlled with rudders spanning the height of the winglets. The current design of the control surfaces has been tested by pilots in a simulator [2], in which the aerodynamic model is used to describe the aircraft relied solely on vortex lattice method simulations combined with wind tunnel data to partially consider the pitch break occurring at an angle of attack of approximately 20$^\circ$. The tests showed an unstable Dutch roll response of the aircraft, and to test the requirements outlined by EASA CS-25 [7] the lateral-directional dynamics were decoupled from the longitudinal dynamics. The Dutch roll response of the aircraft is discussed in Sect. 2.6.

Furthermore, the simulator tests featured various maneuvers as the ones tested in this work and outlined in Sect. 2.1. The tests showed a lack of rudder’s control authority and some slight pitch dropout during the bank-to-bank maneuver due to elevator saturation. Most longitudinal results were between level 1 and level 2. This classification is achieved by converting Cooper–Harper ratings into handling quality levels from 1 to 3, as outlined in the equivalency chart defined in the military standard 1797 A [14]. Overall, the simulation tests showed that the design of control surfaces should be improved to meet all certification requirements, and a more complex aerodynamic model would be required to identify nonlinearities occurring in the various flight phases.

Conventional aircraft employ the aileron, elevator, and rudder to control and maneuver the aircraft. However, alternative control devices could serve similar purposes, potentially being more effective for aircraft like the Flying-V. For example, split drag-rudders can create a yawing moment by creating a drag difference between the left wing and the right wing [17] - [20]; spoilers located on the inner part of the wing can also induce yawing and rolling moments [16]. To limit the scope of this work, only aileron, elevator, and rudder are considered in this study. However, if certification requirements cannot be met, it is stipulated whether these alternative control surfaces could potentially be used to solve this problem.

In this study, the maneuvers are simulated offline, i.e., not in the flight simulator. This allows the testing of new control surface layouts with low workload and computational efforts. Previous studies have sized control surfaces similarly based on offline handling quality evaluations in unconventional aircraft [6]-[19]. A similar approach is also pursued in this paper, with an aerodynamic model of the aircraft which incorporates Reynolds-averaged Navier–Stokes (RANS) simulations and hence nonlinear effects in the early phase of the control surface sizing process [1].

The contribution of this paper is to size, position, and define the control surfaces required for a Flying-V aircraft. The control surface layout is driven by the maneuvers that the Flying-V must be able to perform inside its flight envelope. The maneuvers are defined by handling quality requirements, and compliance is demonstrated through offline simulations.

The aerodynamic model of the Flying-V is introduced in Sect. 2.2, together with the flight conditions considered. The maneuvers tested are presented in Sect. 2.1. For each maneuver, the required control authority of the elevator, the aileron, and the rudder is estimated, and then, the control surfaces are each sized and placed on the wing accordingly. Their combination defines the control surface layout for the Flying-V aircraft.

## Methodology

The control surfaces are sized according to the maneuvers described in Sect. 2.1. The maneuvers are simulated in a framework in which the aerodynamic model (Section 2.2) and the rigid-body dynamics of the aircraft (Section 2.3) are integrated. For each maneuver, simulations are run iteratively to identify a range of control surface derivatives that can satisfy its requirements. The minimum control surface derivative, in absolute value, is then selected as the sizing case and is used to find a control surface on the aircraft that can provide the requested control surface derivative. The same process is then repeated for all the selected maneuvers, leading to the new control surface layout.

### Criteria for sizing control surfaces

The aileron, the elevator, and the rudder control the moment around the body *x-*, *y-*, and *z*-axes, respectively. The moments around the three axes are here indicated as $C_\text {l}$, $C_\text {m}$, and $C_\text {n}$. The body axis reference frame has its origin at the Center of Gravity (CG) which is defined in Sect. 2.3.

In the certification documentation [7], a series of criteria are defined that allow the sizing of the control surface required for each function. Particularly, the elevator for pitch control, the aileron for roll control, and the rudder for yaw control. However, the control surface on the trailing-edge area can potentially act as both elevator and aileron, i.e., an elevon. To discriminate between high-rate and low-rate actuators which have a direct impact on the power consumption and the weight, the elevon is sized to provide the required roll control, and if the elevon cannot provide enough pitch control an additional control surface is designed to serve as a pure elevator and it is located more inboard than the elevon.

Herein, the functions used to size the different control surfaces are summarized:

#### Elevator

- *Longitudinal trim*: the elevon and the elevator are sized to longitudinally trim the aircraft. The procedure to distinguish between elevon and elevator is discussed in detail in Sect. 3.1. The longitudinal trim procedure allows determining the required angle of attack $\alpha$ and thrust *T* at a certain flight condition. The identified longitudinal trim condition is then used as the starting point for the remaining cases. Furthermore, the thrust lapse with the operating conditions is estimated for the engine considered in this work, for which the sea-level static thrust-to-weight ratio is $T_\text {SLS}/W_\text {MTOW} = 0.27$. A detailed description of the engine can be found in [10]. The thrust is assumed to scale with the Mach number and the altitude according to [13] as
  1 $$\begin{aligned} T_\text {max} = T_\text {SLS} \frac{p_{t, \infty }}{p_0} \left[ 1-(0.43+0.014 \cdot \text {BPR})\sqrt{M_\infty }\right], \end{aligned}$$
  where $p_{t, \infty }$ is the freestream total pressure at cruise altitude, $p_0$ the sea-level pressure of 101 kPa, and $\text {BPR = 10}$ is the bypass ratio of the engine. For the operating conditions considered in this paper, ${T_{t, \infty }}$/${T_0}$ is always lower than 1.06, and hence, Eq. 1 is sufficient to describe the thrust lapse. The total pressure and temperature are calculated with the isentropic equations. The influence of the CG on the control surface size is described in Section 2.3 and the trimming algorithm is discussed in Section 2.5.
- *Pull-up and push-over maneuver*: the aircraft has to pull-up to at least 1.5*g* and push-over to 0.5*g* at the different flight conditions. The limits are reduced to 1.3*g* and 0.5*g* at landing speed.
- *Take-off rotation*: the aircraft is supposed to provide a rotation rate of 3$^\circ$/s at rotation speed and Maximum Take-off Weight.

#### Rudder

- *Steady-heading sideslip*: The aircraft should keep a constant heading while facing a certain sideslip angle $\beta$. This maneuver assesses the capabilities of the aircraft during crosswind. The certification documentation EASA CS-25 (AMC 25.177(c)) [7] defines $\beta = \text {asin}(30/U_\infty )$, where the velocity is expressed as calibrated airspeed in knots. The sideslip values in absolute value are listed in Table 1 for the flight conditions considered here.
- *One-engine-inoperative*: A second function of the rudder is to compensate the $C_\text {n}$ induced by one engine when the other engine fails. Although the precise location of the engines is still under investigation [10], it is assumed to be located at *y* = 5.7 m, *z* = 0 m. Furthermore, it is tested the one-engine-inoperative case in combination with the crosswind cases.

#### Aileron

- *Bank-to-bank*: The aircraft is required to bank from −30$^\circ$ to 30$^\circ$ in 7 s or less. During this maneuver, the flight path angle should be kept between 0$^\circ$ and 5$^\circ$.

**Table 1:** Sideslip angle $\beta$ at the considered flight conditions

$M_\infty$	0.2	0.25	0.3	0.4	0.6	0.7	0.85
$\beta$	13.1$^\circ$	10.5$^\circ$	8.7$^\circ$	8.9$^\circ$	7.0$^\circ$	6.9$^\circ$	6.2$^\circ$

Additionally, the aileron is also used to compensate for the rolling moment occurring during the one-engine inoperative and steady-heading-sideslip maneuvers.

#### Coordinated turn

After sizing the control surfaces, a coordinated turn is considered to combine longitudinal and lateral–directional motions. This maneuver allows testing the combination of the three control surfaces, i.e., studying the aircraft’s capability to coordinate yaw, pitch, and roll simultaneously. A turn is performed at a constant speed while minimizing the sideslip angle. The requirement is to perform the maneuver for 10 s while keeping the bank angle between 40$^\circ$ and 45$^\circ$ and the flight path angle between 0$^\circ$ and 5$^\circ$, to prevent altitude loss. In the worst case scenario with a flight path angle of 5$^\circ$ and the highest speed tested in this work (Table 3), the change in altitude corresponds to 219 m which does not invalidate the operating condition tested because of the limited change in the aerodynamic coefficients.

### Aerodynamic model

The parameters influencing the aerodynamic forces and moments are determined with a combination of Reynolds-averaged Navier–Stokes (RANS) and Vortex Lattice Method (VLM) simulations. These models are thoroughly discussed in [1]. A summary of the parameters that influence the forces and moments acting on the aircraft is presented in Table 2, which also indicates if the parameter is determined with RANS (R) or VLM (V). The variables in this table are the angle of attack ($\alpha$), the sideslip angle ($\beta$), the elevator deflection ($\delta _\text {e}$), the aileron deflection ($\delta _\text {a}$), the rudder deflection ($\delta _\text {r}$), and the high/lift flap deflection ($\delta _\text {f}$). Furthermore, it contains the dimensionless angular rates, i.e. the roll rate ($\hat{p} = pb/(2U_\infty )$), the pitch rate ($\hat{q} = q\overline{c}/(2U_\infty )$), and the yaw rate ($\hat{r} = rb/(2U_\infty )$), where *p*, *q,* and *r* are the angular rates with respect to the *x-*, *y-,* and *z*-axes, respectively.

**Table 2:** Force and moment coefficient dependencies, marked with “R” when determined through RANS simulations and with “V” when determined through VLM simulations

	$\alpha$	$\beta (\alpha )$	$\hat{p}$	$\hat{q}$	$\hat{r}$	$\delta _\text {e} (\alpha )$	$\delta _\text {a} (\alpha )$	$\delta _\text {r} (\alpha )$	$\delta _\text {f} (\alpha )$
$C_{x}$	R					V - R	V - R	V - R	V - R
$C_{y}$		R			V		V - R	V - R	
$C_{z}$	R			V		V - R			V - R
$C_\text {l}$		R	V		V		V - R	V - R	
$C_\text {m}$	R			V		V - R			V - R
$C_\text {n}$		R	V		V		V - R	V - R	

The RANS simulations determine the forces and moments generated by a single control surface design for the aileron, elevator, high-lift flap, and rudder. The results are then used to calibrate the VLM simulations, which are less time-consuming. For this reason, the results presented in Sect. 3, are divided into "VLM" and "VLM calibrated with RANS". More details on the control surfaces simulated with RANS are introduced in [1].

**Fig. 2:**
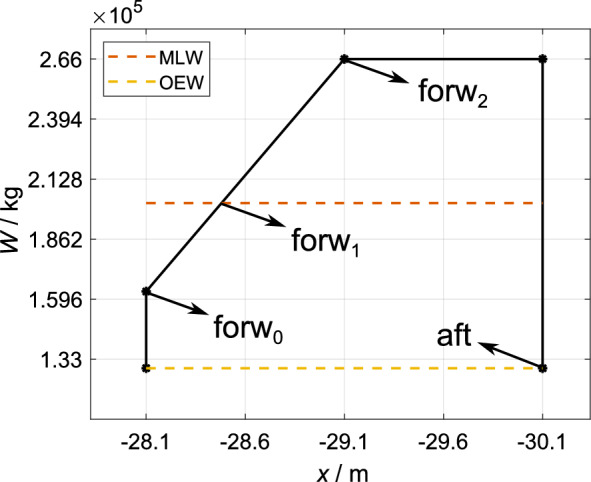
CG range of the aircraft and CG locations highlighted

The maneuvers, presented in Section 2.1, are tested at several flight conditions, as summarized in Table 3. The selected operating conditions are chosen to represent different phases of the flight envelope. The different conditions are divided into two groups, those at maximum landing weight (MLW) where $m_\text {MLW}$ = 202$\cdot$10$^3$ kg, and those at maximum take-off weight (MTOW) where $m_\text {MTOW}$ = 266$\cdot$10$^3$ kg. The masses are derived from [15], assuming a family-optimized Flying-V-1000 aircraft. The relationship between the CG range and the weights is depicted in Fig. 2. The control surface sizing is conducted for the most forward CG location of the aircraft indicated as forw$_0$. A detailed description of the CG diagram can be found in [1].

**Table 3:** Flight conditions

	*h*/m	$\rho _\infty$/(kg/m$^3$)	$U_\infty$/(m/s)	$M_\infty$	$Re_\text {c}$/10$^7$	*m*/(10$^3$ kg)
MLW	0	1.22	68	0.20	8.4	202
	0	1.22	85.1	0.25	10	202
	0	1.22	102.1	0.30	13	202
MTOW	0	1.22	102.1	0.30	13	266
	5450	0.70	127.5	0.40	10	266
	7650	0.55	185.7	0.60	12	266
	9750	0.43	210.4	0.70	11	266
	11225	0.35	250.8	0.85	11	266

As discussed above, three different types of control surfaces are considered: elevator, elevon, and rudder. Table 4 indicates their maximum and minimum deflection angles and maximum deflection rates, based on [9]. The indicated limits are used during the simulations. However, new maximum and minimum deflections are suggested in Section 3 to satisfy specific requirements when needed.

**Table 4:** Control surfaces' characteristics

	$\delta$/$^\circ$	$\dot{\delta }$/($^\circ$/s)
Elevator	± 25	40
Aileron/elevon	± 25	55
Rudder	± 25	50

### Rigid-body dynamics

The aerodynamic force and moment coefficients are expressed in the body reference frame and converted into linear and rotational accelerations. The system of equations is derived based on five assumptions: the aircraft is rigid and has a constant mass *m*, the Earth is flat and non-rotating, the gravitational acceleration *g* is constant, the aircraft has a plane of symmetry in the body-fixed reference frame which leads to $I_\text {xy} = I_\text {yz} = 0$, and finally, when both engines are operative, the resultant thrust vector is on the symmetry plane and hence only affects the force along *x*. Based on these assumptions, the system of equations can be expressed as follows:

2 $$\begin{aligned} \begin{aligned} A_x&= \frac{C_{x}}{m} - g\sin \theta \\ A_y&= \frac{C_{y}}{m} + g\sin \phi \cos \theta \\ A_z&= \frac{C_{z}}{m} + g\cos \theta \cos \phi \\ \dot{p}&= \frac{I_{zz}}{I^*}C_\text {l} + \frac{I_\text {xz}}{I^*}C_\text {n} + \frac{\left( I_{xx}-I_{yy}+I_{zz}\right) I_\text {xz}}{I^*}pq \\&\quad + \frac{\left( (I_{yy}-I_{zz})I_{zz} - I^2_\text {xz}\right) }{I^*}qr\\ \dot{q}&= \frac{C_\text {m}}{I_{yy}} + \frac{I_\text {xz}(r^2-p^2)}{I_{yy}} + \frac{I_{zz}-I_{xx}}{I_{yy}}pr\\ \dot{r}&= \frac{I_\text {xz}}{I^*}C_\text {l} + \frac{I_{xx}}{I^*}C_\text {n} + \frac{\left( -I_{xx}+I_{yy}-I_{zz}\right) I_\text {xz}}{I^*}qr \\&\quad + \frac{\left( (I_{xx}-I_{yy})I_{xx} + I^2_\text {xz}\right) }{I^*}pq,\\ \end{aligned} \end{aligned}$$

where $I^* = I_{xx}I_{zz} - I_\text {xz}^2$. All linear and angular body velocities can be obtained by integrating these equations. In addition, the kinematic equations that allow converting body velocities into attitude angles can be expressed as follows:

3 $$\begin{aligned} \begin{aligned} \dot{\phi }&= p + q\sin \phi \tan \theta + r\cos \phi \tan \theta \\ \dot{\theta }&= q\cos \phi - r\sin \phi \\ \dot{\psi }&= q\frac{\sin \phi }{\cos \theta } + r\frac{\cos \phi }{\cos \theta }. \end{aligned} \end{aligned}$$

The moments and products of inertia of the aircraft used in Eq. 2 are summarized in Table 5. The inertia terms are calculated at maximum take-off (MTOW) and maximum landing (MLW) weights, for each weight they are calculated at the most forward (forw$_1$ for MLW and forw$_2$ for MTOW) and aft Center of Gravity (CG) positions. The location of the different CG considered are summarized in Fig. 2. Because of the presence of the plane of symmetry, $I_\text {xy} = I_\text {yz}$ = 0.

The inertia terms are calculated by dividing the aircraft mass into 100 point masses along the span, being located at the mid-chord of each section. The masses distribution along the aircraft is detailed in [18], where higher masses are used in the inner zone to indicate the passenger and cargo contribution as in Fig. 1b. The mass distribution is then adjusted to obtain the required CG location, by employing a pumping system to distribute the kerosene between the different fuel tanks [18]. As expected for both weights, when moving from forward to aft CG location, the inertia term increases in absolute value. The increase is higher for the MLW case, because the CG range is higher than at MTOW.

**Table 5:** The moments and products of inertia at different aircraft weights and CG locations

	$I_{xx}$/	$I_{yy}$/	$I_{zz}$/	$I_\text {xz}$/	($I_\text {xy} = I_\text {yz}$)/
	(kg$\cdot$m$^2$)	(kg$\cdot$m$^2$)	(kg$\cdot$m$^2$)	(kg$\cdot$m$^2$)	(kg$\cdot$m$^2$)
MLW forw$_1$ CG	3.4$\cdot$10$^7$	2.9$\cdot$10$^7$	6.2$\cdot$10$^7$	−0.09$\cdot$10$^7$	0
MLW aft CG	4.1$\cdot$10$^7$	3.2$\cdot$10$^7$	7.2$\cdot$10$^7$	−0.11$\cdot$10$^7$	0
MTOW forw$_2$ CG	4.8$\cdot$10$^7$	3.9$\cdot$10$^7$	8.7$\cdot$10$^7$	−0.13$\cdot$10$^7$	0
MTOW aft CG	5.3$\cdot$10$^7$	4.2$\cdot$10$^7$	9.5$\cdot$10$^7$	−0.14$\cdot$10$^7$	0

The impact of the moments and products of inertia on the Dutch roll characteristics of the aircraft is considered in Section 2.5. For the control surface sizing in Section 3, initial tests show a limited impact of the inertia, with the CG location being the main driver. For a more conservative design, the most forward CG location of the aircraft at any weight (forw$_0$) is considered for the sizing process of the control surfaces.

### Actuator and engine dynamics

The control surfaces or actuators (act) are modeled as a second-order system based on [12], and the engines (eng) are modeled as a first-order system. Their transfer functions are as follows:

4 $$\begin{aligned}&H_\text {act}(s) = \frac{4000}{s^2 + 140s + 4000} = \frac{\omega ^2_\text {n}}{s^2 + 2\zeta \omega _\text {n}s + \omega ^2_\text {n}}\nonumber \\&\quad \text {and}\hspace{0.4cm} H_\text {eng}(s) = \frac{1}{0.2s+1}. \end{aligned}$$

For the actuators, this leads to a natural frequency $\omega _\text {n} = 63.25$ rad/s and a damping ratio $\zeta = 1.11$.

### Simulation model

**Fig. 3:**
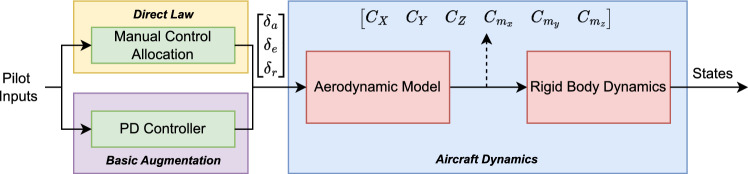
Model structure for offline simulations

Figure 3 shows the layout of the simulation model, which encapsulates the aerodynamic model, the rigid-body dynamics, and the control law used to command the aircraft.

**Fig. 4:**
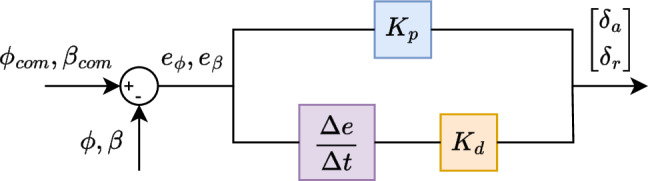
PD controller used in basic augmentation

Based on the figure, two types of control laws act as bridges between the pilot commands and control surface deflections. In direct law, the pilot inputs are manually mapped over the control surfaces, such that the pilot’s sidestick and pedal inputs directly command the control surfaces. Normally, this would be sufficient to study the aircraft’s handling qualities in a real-time simulation environment. However, since simulations are conducted offline, a basic automatic control augmentation is necessary to perform maneuvers without a pilot. It is designed to have a proportional-derivative structure. The control deflections computed through either direct law or basic augmentation serve as inputs to the aerodynamic model to calculate the forces and moments acting on the aircraft. These are then used in dynamical equations given in Equation 2. The final states of the aircraft are calculated by integrating the linear and rotational accelerations of the aircraft, and using the kinematic transformations given in Equation 3. The structure of the PD controller is shown in Fig. 4.

When the controller is active, it is applied only to the roll and sideslip channels. Several maneuvers in this study require the aircraft to reach and maintain a specific sideslip and/or bank angle. The pitch channel uses the direct law in all cases, controlling the symmetric deflection of the elevons, which corresponds to an elevator input. The resulting motion causes changes in the angle of attack, pitch angle, flight path angle, and altitude. The gains of the controller are obtained by trial and error, separately for roll and sideslip, resulting in $K_{\text {p}_\beta } = K_{\text {d}_\beta } = K_{\text {p}_\phi } = 5000$ and $K_{\text {d}_\phi } = 1000$. The trial-and-error process aims to find a fast control response without excessive overshoot. Although the gains might seem high at first glance, this is because the sideslip and beta are defined in radians, whereas the aileron and rudder deflections are in degrees.

For the sizing maneuvers explained in Section 2.1, the controller is active for steady-heading sideslip, one-engine-inoperative, bank-to-bank, and coordinated turn. The longitudinal trim condition is calculated statically using a cost function and minimization algorithm. The direct law is used without any control augmentation to test the calculated trim point in a steady-level flight scenario. The aim of the cost function and the minimization algorithm is to find a steady state for the aircraft such that the longitudinal acceleration terms, $\dot{u}$, $\dot{w}$, $\dot{q}$ and the flight path angle $\gamma$ are zero. In addition, the cost function seeks to minimize the difference between the desired airspeed ($U_\text {des}$) and the trim airspeed ($U_\text {trim}$). This cost function is given as follows:

5 $$\begin{aligned} & J(\dot{u},\dot{w},\dot{q},\dot{V},\dot{\gamma }) = 100\cdot \dot{u}^2 + 1\cdot \dot{w}^2 \nonumber \\ & \quad + 100\cdot \dot{q}^2 + 100\cdot (U_\text {des}-U_\text {trim})^2 + 100\cdot \gamma ^2. \end{aligned}$$

The variables of this cost function can all be expressed in terms of aerodynamic forces and moments using Equation 2. Hence, the problem of minimizing the accelerations indirectly becomes a net force/moment minimization problem. Given that longitudinal forces and moments are related to elevator deflection $\delta _\text {e}$, angle of attack $\alpha$, and engine thrust *T*, the minimization algorithm tries to iteratively change these parameters to find the global minimum of the cost function. The algorithm is implemented in Matlab using the "fmincon" function, employing a sequential quadratic programming (SQP) approach.

### Dutch roll analysis

**Fig. 5:**
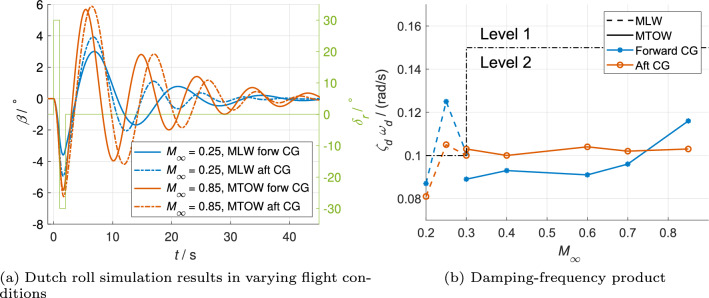
Dutch roll simulation results and damping–frequency product

In a recent simulator flight test conducted on an aerodynamic model of the Flying-V which relied only on VLM simulations, the open loop Dutch roll is found to be unstable [2]. Consequently, this is a point of concern for the new aerodynamic model used in this study. For this reason, as a pre-check before running the actual sizing simulations, an offline Dutch roll simulation is conducted for all flight conditions using the direct law. Control augmentation for the Dutch roll is avoided to prevent masking the fundamental response of the aircraft. To this end, the nonlinear simulation model is excited using a doublet rudder input and then allowed to evolve without interference to observe whether the sideslip oscillations dampen out over time. In Dutch roll simulations, the aircraft is initialized from a trimmed steady-level condition, where it stays for 10 s until the rudder doublet excites the lateral–directional dynamics and the simulation is then run for 300 s. Figure 5a shows the rudder input varying between $\delta _\text {r}$ = ± 30$^\circ$, and the time histories of the sideslip angles at two different operating conditions, $M_\infty$ = 0.25 and MLW, and $M_\infty$ = 0.85 and MTOW. For the sake of demonstration, the figure discards the first 10 s of the simulation where the steady flight takes place. Each condition is tested at two CG locations with their related inertia presented in Table 5. Moving from the forward to the aft CG locations at both $M_\infty$, the frequency increases and the oscillations damp out more slowly. To analyze these time histories in more detail, the logarithmic decrement method is used to calculate the natural frequency $\omega _\text {d}$ and the damping ratio $\zeta _\text {d}$ of the oscillations, which are given as follows:

6 $$\begin{aligned} & \omega _\text {d} = \frac{2\pi }{n}\left( \sum _{k = 0}^{n} t_\text {k+1}-t_\text {k} \right) \end{aligned}$$

7 $$\begin{aligned} & \zeta _\text {d} = \frac{1}{n}\log \left( \sum _{k = 0}^{n} \frac{x(t_\text {k})}{x(t_\text {k+1})} \right), \end{aligned}$$

where *n* is the number of peaks, *x* is the amplitude of the peaks, and $t_\text {k}$ is the time at which the peaks occur. Hence, the damping ratio is calculated by taking the sum of consecutive peaks and averaging them, whereas the frequency comes from averaging the consecutive periods of oscillation. $\omega _\text {d}$ and $\zeta _\text {d}$ are calculated considering the peaks with positive sideslip angle.

The final values of $\zeta _\text {d}$ and $\omega _\text {d}$ determine the handling qualities based on the MIL-STD-1797A standards [14]. Assuming that the Flying-V model falls under class III aircraft, the classifications in Table 6 are considered for the terminal and nonterminal flight phases. An important point to mention is that these Dutch roll classifications are mainly used for linearized aircraft models. However, one could argue that using a nonlinear simulation model is more accurate and allows for the possibility of revealing undesired phenomena. Nevertheless, due to the nonlinear nature of the model, the handling quality ratings for the Dutch roll serve as an approximation.

**Table 6:** Dutch roll classifications based on MIL-STD-1797A

	Category C - terminal flight phases			Category B - nonterminal flight phases		
	$\omega _\text {d}$/(rad/s)	$\zeta _\text {d}$	$\zeta _\text {d} \omega _\text {d}$/(rad/s)	$\omega _\text {d}$/(rad/s)	$\zeta _\text {d}$	$\zeta _\text {d} \omega _\text {d}$/(rad/s)
Level 1	$\ge$ 0.4	$\ge$ 0.08	$\ge$ 0.10	$\ge$ 0.4	$\ge$ 0.08	$\ge$ 0.15
Level 2	$\ge$ 0.4	$\ge$ 0.02	$\ge$ 0.05	$\ge$ 0.4	$\ge$ 0.02	$\ge$ 0.05
Level 3	$\ge$ 0.4	$\ge$ 0	-	$\ge$ 0.4	$\ge$ 0	-

**Table 7:** Dutch roll analysis and classification results

Flight conditions	Flight phase	CG	$\omega _\text {d}$/(rad/s)	$\zeta _\text {d}$	$\zeta _\text {d} \omega _\text {d}$/(rad/s)	HQ level
M = 0.2 (MLW)	Terminal	forw$_1$	0.42	0.20	0.087	2
		aft	0.47	0.17	0.081	2
M = 0.25 (MLW)	Terminal	forw$_1$	0.45	0.27	0.125	1
		aft	0.82	0.13	0.105	1
M = 0.3 (MLW)	Terminal	forw$_1$	0.46	0.22	0.101	1
		aft	0.90	0.11	0.100	1
M = 0.3 (MTOW)	Nonterminal	forw$_2$	0.49	0.18	0.089	2
		aft	0.84	0.12	0.103	2
M = 0.4 (MTOW)	Nonterminal	forw$_2$	0.55	0.17	0.093	2
		aft	0.80	0.12	0.100	2
M = 0.6 (MTOW)	Nonterminal	forw$_2$	0.69	0.13	0.091	2
		aft	0.88	0.12	0.104	2
M = 0.7 (MTOW)	Nonterminal	forw$_2$	0.69	0.14	0.096	2
		aft	0.81	0.13	0.102	2
M = 0.85 (MTOW)	Nonterminal	forw$_2$	0.70	0.17	0.116	2
		aft	0.77	0.13	0.103	2

Using Equations 6 and 7, and comparing the results with the Dutch roll requirements, it can be observed that the Dutch roll is stable and damped under all flight conditions. The results are summarized in Fig. 5b and Table 7. The figure only depicts the damping-frequency product, because it serves as the primary driving factor for the Handling Qualities (HQ) classifications in this application. For $M_\infty \ge$ 0.3 and MTOW, Fig. 5b shows that the Dutch roll is within Level 2. At MLW, the Dutch roll is at Level 1 for $M_\infty$ = 0.25 and 0.3, and at Level 2 for $M_\infty$ = 0.2. As will be discussed in Section 3, at $M_\infty$ = 0.2, a high-lift flap could be required, and when simulating the Dutch roll of high-lift configuration, the HQ diminishes to Level 3 at the forward CG and drops below 3 at the aft CG. Further investigations are underway to identify the source of the drop in HQ level.

With MLW at the forward CG, the aircraft is more stable than at the aft CG. Differently, the opposite occurs at MTOW with the exception of $M_\infty$ = 0.85. Table 7 shows that for all the cases, $\zeta _\text {d}$ decreases and vice versa $\omega _\text {d}$ increases when moving from forward to aft CG. The main trend that can be observed with increasing $M_\infty$ is that $\omega _d$ increases at the aft CG location. Additionally, some differences in trends can be noticed between subsonic and high subsonic/transonic cases as it will also be seen in Section 3.

## Results

Section 3.1 introduces how the initial trim conditions are obtained that are required for all subsequent simulations. Section 3.2 presents the aileron dimensions and deflection needed to satisfy the certification requirements. Subsequently, the sized aileron is also assigned a pitch control function, making it an elevon. If this elevon is unable to satisfy a pitch control requirement by itself, an additional elevator is sized next to the elevon on the inboard side (Section 3.3). Section 3.4 presents the sizing of a high-lift flap and discusses why it is needed at low speed. Section 3.5 investigates the dimension and deflection of the rudder to satisfy the certification requirement. The newly defined control surfaces are tested with the coordinated turn in Section 3.6. Finally, Section 3.7 defines the stall speed and related speeds as the rotation speed, indicating which are the maneuver that the aircraft can perform at these speeds.

### Initial trim conditions

An elevator is initially sized to find longitudinal trim conditions. The trim conditions are required to define the angle of attack $\alpha$ and the thrust *T*, which are needed for all the remaining simulations.

**Fig. 6:**
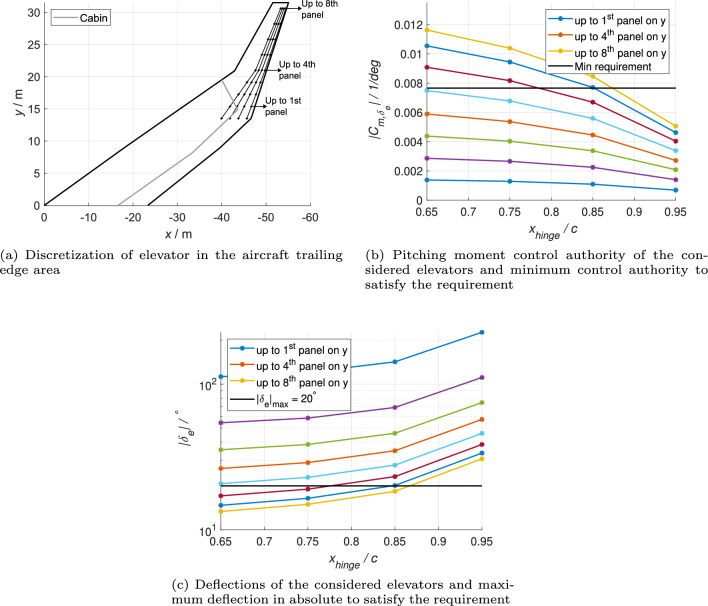
Discretization of the elevator in the trailing-edge area, and corresponding pitching moment coefficient and related deflection due to elevator for trimming

Figure 6a shows the discretization of the outer wing trailing-edge area used to test different elevator designs. The considered area is discretized into eight panels along the span and four along the chord. Initially, for the elevator sizing, the most inboard sections are considered to leave the outer sections to place the aileron. For example, the first elevator considered is the most inboard panel with the hinge line closer to the trailing edge, $x_\text {hinge}/c$ = 0.95. After that, the hinge line is moved upstream, up to $x_\text {hinge}/c$ = 0.65. The control authority of the four different described elevators is shown in Fig. 6b as the blue line closer to zero in terms of $C_{\text {m},\delta _\text {e}}$. The same procedure is then applied when increasing the number of panels along the span where each panel has an extension of 2.8%*b*, and with the simplification that the hinge line is fixed, to avoid steps in the control surface or “hollowed” control surfaces. Figure 6b shows the control authority of the considered elevators, which are 32 in total, and Fig. 6c shows the corresponding deflection angle required to satisfy the considered trim condition.

The black horizontal line in Fig. 6b indicates the minimum requirement in absolute value to satisfy the longitudinal trim conditions. This requirement is determined in the different flight conditions introduced in Table 3, with a maximum possible deflection of 20$^\circ$ as shown in Fig. 6c, the limit is lower than in Table 4 to avoid saturation of the control surface for other maneuvers. The minimum requirement is determined by running ten simulations introduced in Section 2.5 and selecting the smaller control authority that can satisfy the deflection angle constraint. A similar procedure is then repeated with the other control surfaces for the selected maneuvers.

**Fig. 7:**
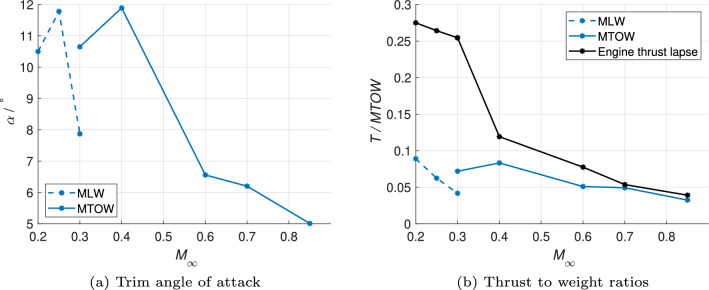
Angle of attack and thrust required for longitudinal trim at various flight conditions and at $x_{\text {CG}}$ = forw$_0$

The output of the longitudinal trim simulations is also the angle of attack $\alpha$ and the thrust *T* as depicted in Fig. 7a and b, respectively. As expected, at fixed weight, $\alpha$ tends to decrease with $M_\infty$, because the dynamic pressure increases. However, at MTOW, this is not the case between $M_\infty$ = 0.3 and 0.4, because also the altitude is varied between the two cases, leading to a reduction of density, which is higher than the increase due to the freestream velocity squared. At MLW, also between $M_\infty$ = 0.2 and 0.25, $\alpha$ increases, in this case, because a high-lift flap is used as it will be discussed in Section 3.4.

The thrust-to-weight ratio is shown in Fig. 7b, where the weight is the maximum take-off weight (MTOW). *T* is the required trim-thrust for level flight, i.e., to compensate the component of the weight and the force along the *x* axis due to $\alpha$ and $\delta _\text {e}$. The elevator considered here is the final one sized in this work. Figure 7b considers the engine introduced in Section 2.1.1. The results show that the thrust available is at least 8% higher than the thrust required.

$\alpha$ and *T* are the initial conditions for the remaining simulations and they are kept constant for all of them. The center of gravity (CG) used in all the considered simulations is located at 28.1 m from the aircraft nose (Fig. 9) and indicated as forw$_0$ (Fig. 2), corresponding to the most forward CG location considered for this aircraft. The combination of MTOW and the most forward CG location of the aircraft represents an extreme combination that would likely not occur in reality, as it is shown in Fig. 2.

### Aileron sizing

As introduced in Section 2.1.3, the aileron is required for three maneuvers. First, during the bank-to-bank maneuver, where the aircraft has to bank from −30$^\circ$ to 30$^\circ$ in 7 s or less. Second, to maintain a bank angle of ±2$^\circ$ during the steady-heading sideslip maneuver. Finally, to compensate for the rolling moments induced by the rudders when the aircraft faces the one-engine-inoperative condition (Section 2.1.2). In this scenario, the rudders help maintain a sideslip angle of ±2$^\circ$, while the ailerons ensure wings-level flight.

**Fig. 8:**
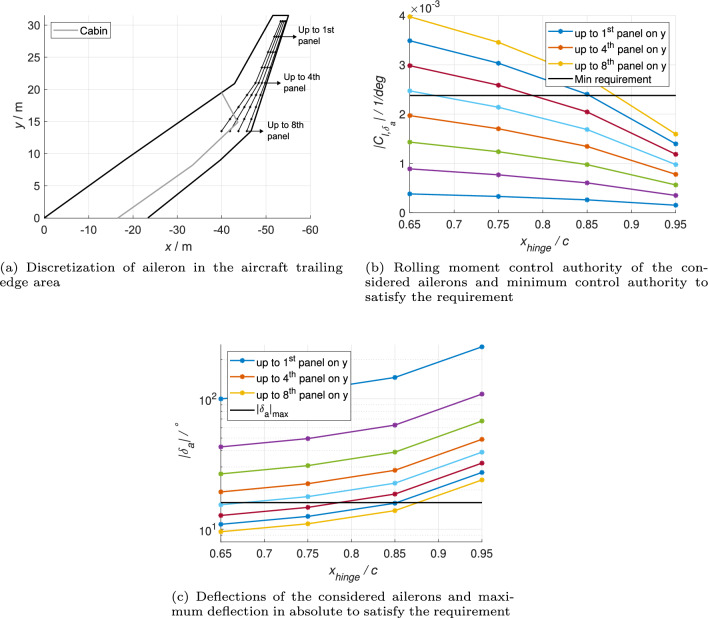
Discretization of the aileron in the trailing-edge area, and corresponding rolling moment coefficient and related deflection due to aileron for sideslip requirement

Starting from the trim conditions identified in Section 3.1, the aileron is sized with a procedure similar to the initial elevator sizing, but differently than for the elevator the aileron sizing starts from the aircraft tip moving inboard (Section 3.1). Figure 8a shows the discretization of the trailing-edge area, Fig. 8b shows the rolling moment generated by each aileron considered, and Fig. 8c shows the corresponding deflection required to satisfy the sideslip requirement at low speed which is the most demanding for the aileron. The last two figures include the minimum requirement in absolute value in term of $C_{l,\delta _a}$ which corresponds to a maximum deflection of 15$^\circ$.

**Fig. 9:**
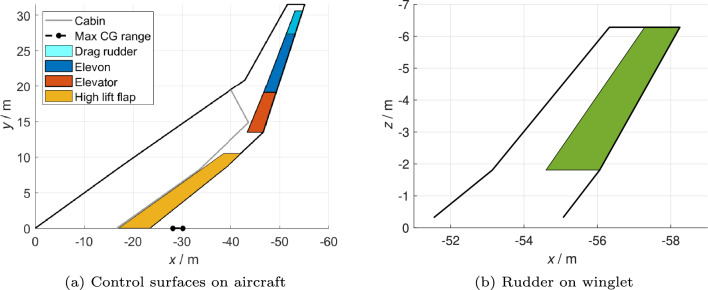
Control surfaces sized in this study positioned on the aircraft. The drag rudder is part of the elevon

The final aileron geometry considered is depicted in Fig. 9, where the aileron is located in the outer part of the aircraft. In the figure, the surface here sized is labeled as elevon, because it serves both as elevator and aileron, but its dimension is driven by the requirement for the aileron.

**Fig. 10:**
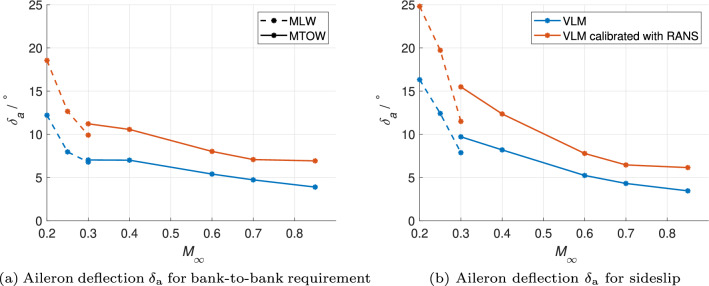
Maximum aileron deflections for bank-to-bank and steady-heading sideslip requirements at various flight conditions and at $x_{\text {CG}}$ = forw$_0$

The deflections required from the aileron for the bank-to-bank maneuver and steady-heading sideslip maneuver are shown in Fig. 10a and b, respectively. The results for the one-engine-inoperative condition are not included for the aileron, because the deflections are much lower than for the depicted cases, being at most $\delta _\text {a}$ = 1.1$^\circ$ at $M_\infty$ = 0.2.

Figure 10a and b comprises the deflection required in the various flight conditions introduced in Table 3. Bold lines indicate MTOW and dashed lines MLW. Additionally, for all the cases, the deflections are calculated with VLM and with VLM calibrated with RANS, as introduced in Section 2.2. Particularly, the aileron is sized so to obtain a maximum deflection of 25$^\circ$, which occurs at $M_\infty$ = 0.2, MLW, $x_\text {CG}$ = forw$_0$, with a high-lift flap deflection of $\delta _\text {f}=40^\circ$ to satisfy the sideslip requirement. Further details on the high-lift flap can be found in Section 3.4.

The difference between the VLM and VLM calibrated with RANS results is related to the different scaling applied to take into account the $\alpha$ and Mach effects. For both VLM and RANS simulations, the increase of $\alpha$ leads to a decrease in control authority. Conversely, the increase in $M_\infty$ leads to an increase in control authority for VLM due to the compressibility correction, while for RANS, the change in control authority is nonlinear due to the formation of shocks and local flow separation [1]. As a result, the deflections for the aileron predicted by the VLM are between 31 and 44% lower than those for the VLM-calibrated-with-RANS. This difference is mainly due to the higher reduction in control authority with $\alpha$ as predicted by RANS.

It should be noted that, as introduced in Section 2.2, the forces and moments due to $\alpha$ and $\beta (\alpha )$ are always calculated with RANS simulations. Hence, if the aerodynamic model had been derived only with VLM simulations, the obtained deflections could have been even further from the VLM-calibrated-with-RANS simulations.

As mentioned before, the most critical condition, in terms of deflection, for the aileron occurs at $M_\infty$ = 0.2 during the approach; being for the sideslip maneuver $\delta _\text {a} = 25^\circ$, and for the bank-to-bank maneuver, $\delta _\text {a} \simeq <span class='crossLinkCiteEqu'>19</span>^\circ$. This is in line with the aileron deflection limits implemented in modern aircraft.

Up to $M_\infty$ = 0.4, the aileron deflection required during a steady-heading-sideslip maneuver is higher than the one required for the bank-to-bank maneuver. Differently, for $M_\infty \ge 0.6$, the bank-to-bank maneuver requires higher deflections.

### Elevator sizing

The aileron sized in Section 3.2 can also be employed as a pitch control device. Therefore, from now on, the aileron is used as an elevon. However, the moment created by this elevon is not sufficient to trim the aircraft at low speed. Therefore, the elevator initially sized in Section 3.1 is resized to provide the additional elevator power required and it is located next to the elevon on the inboard side, keeping the same chord ratio at the interface between the two control surfaces, as depicted in Fig. 9.

**Fig. 11:**
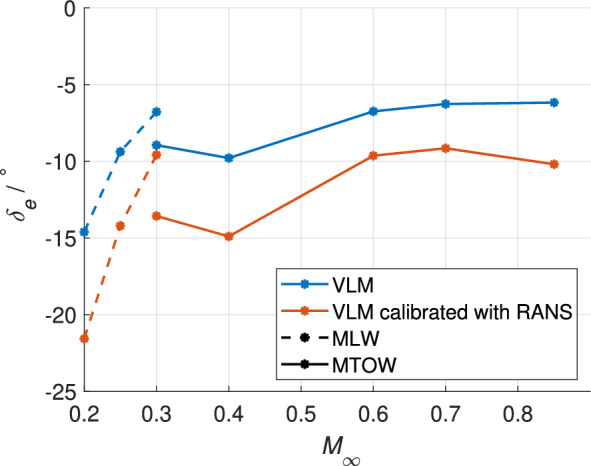
Elevator and elevon trim deflection angles at various flight conditions and at $x_{\text {CG}}$ = forw$_0$

Figure 11 shows the deflection required by the selected elevon and the elevator under different flight conditions. As expected, the figure shows that the elevator deflection requirement decreases with $M_\infty$. The maximum deflection occurs at $M_\infty$ = 0.2, MLW, $x_\text {CG}$ = forw$_0$, with a high-lift flap deflection of $\delta _\text {f}=40^\circ$ (discussed in Section 3.4). Similar to the aileron results, the elevator deflections predicted by VLM are between 29% and 39% lower than those predicted by VLM-calibrated-with-RANS simulations.

As introduced in Section 2.1.1, the elevator is also required for the pull-up, push-over, and the rotation maneuvers. The certification specifications require that they should be performed at specified speeds, such as the rotation speed and the landing speed, which will be discussed in Section 3.7, together with the consequent requirements for the elevator.

### High-lift flap

One of the challenges of the planform geometry of the Flying-V is an unstable pitch break at high angle of attack. This limits the effective maximum lift coefficient and therefore determines the stall speed of the airplane. To increase the lift coefficient without increasing the angle of attack of stall, a trailing-edge flap is proposed over the inboard wing of the Flying-V (see Fig. 9. The flap is located inboard of the inner wing, and the position near the supposed CG range minimally impacts the aircraft’s pitching moment. The exact location of the surface along the span and, therefore, the extension along the chord can depend on the location of the engine, which is still under investigation [10]. The high-lift surface has been simulated as a plain flap. However, due to the presence of the fuel tank [15] and to reduce interference with the flow at the engine, the high-lift flap would realistically be installed on the lower side of the aircraft in the form of a split flap, which is actuated similarly to a spoiler. This split flap implementation has been experimentally characterized in [8], proving the ability to increase the lift for a given angle of attack with minimum impact on the pitching moment.

**Fig. 12:**
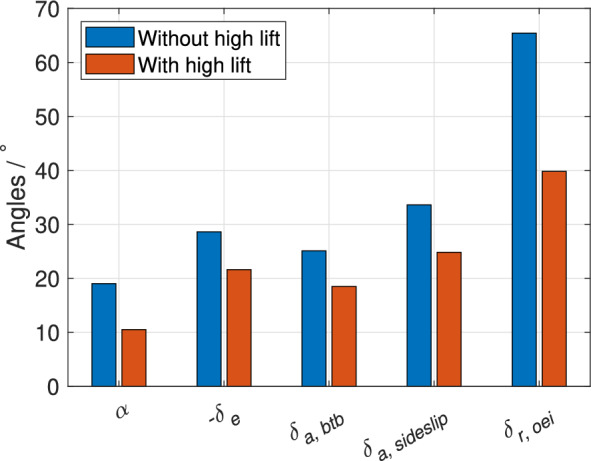
Impact of high-lift flap on the angle of attack and control surface deflections at $M_\infty =0.2$, *h* = 0 m, at $x_{\text {CG}}$ = forw$_0$, and $W=W_\text {MLW}$

Figure 12 shows the effect of the high-lift flap deflected over 40$^\circ$ on the effectiveness of the various control surfaces. As can be seen, the required control surface deflections decrease due to the deployment of the high-lift flap because of the higher effectiveness of the control surfaces at lower angles of attack. The high-lift condition considered herein leads to a reduction in the required deflections of 25% for the elevator, 26% for the aileron, and 39% for the rudder.

As done previously, the VLM is calibrated with RANS simulations, leading to a flap deflection of ca. 40$^\circ$ to obtain the results in Fig. 12. The forces and moment generated by the high-lift flap lead to an 8.5$^\circ$ angle of attack decrease at $M_\infty =0.2$. The size of the flap and its deployment angle have been selected to obtain a $\delta _\text {e} < 25^\circ$ for longitudinal trim and $\delta _\text {a} < 25^\circ$ during the steady-heading sideslip maneuver. In future studies, based on an updated engine location, the high-lift flap will be design such as to create a zero-pitching moment flap at the most forward CG, leading to a smaller requirement for the elevator/elevon.

### Rudder sizing

As introduced in Section 3.5, the rudder is sized to comply with the steady-heading sideslip and one-engine-inoperative conditions.

**Fig. 13:**
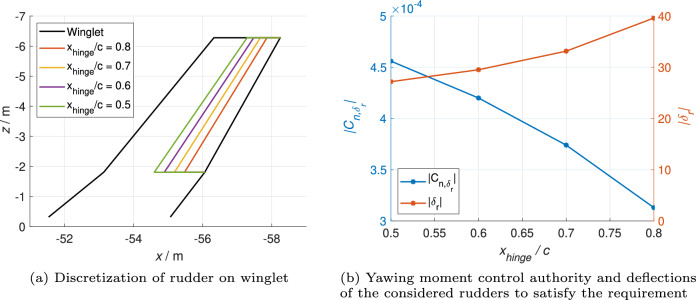
Discretization of the rudder in the winglet, and corresponding yawing moment coefficient and related deflection due to rudder for one-engine-inoperative requirement

The rudder is sized with a similar but simpler procedure with respect to the the other control surfaces considered so far. Figure 13a shows the discretization of the trailing-edge area of the winglet where the span extension of the different rudder is always the same and only the hinge line varies. Figure 13b shows the yawing moment generated by each rudder considered and the corresponding deflection required to satisfy the one-engine-out requirement at low speed which is the most demanding for the rudder. In this case, Fig. 13b does not feature a maximum requirement in terms of deflection or a minimum in term of coefficient, because the maximum possible rudder is considered for the continuation of the study, and the reason is further discussed hereafter.

**Fig. 14:**
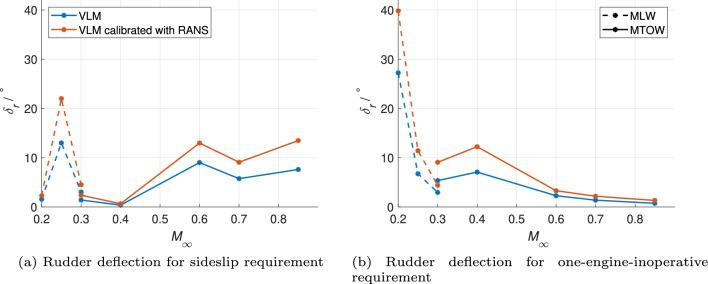
Rudder deflections for sideslip and one-engine-inoperative requirements at various flight conditions and at $x_{\text {CG}}$ = forw$_0$

The sized rudder with $x_\text {hinge}/c$ = 0.5 is depicted in Fig. 9, and the deflections required for the two maneuvers are depicted in Fig. 14.

The maximum rudder deflection in Fig. 14a occurs at $M_\infty$ = 0.25, being 22$^\circ$, which is lower than what is typically achieved by rudders on vertical tails of modern transport aircraft. As for the other control surfaces, the VLM predicts between 30% and 43% lower deflections than VLM calibrated with RANS. As explained in Section 2.2, the force and moment coefficients induced by $\beta (\alpha )$ are always determined with RANS simulations.

Before proceeding, it should be noticed that the sideslip requirements vary with flight conditions as described in Section 3.5. In particular, the maximum value of $\beta$ decreases with increasing freestream velocity and, therefore, with $M_\infty$. However, the required rudder deflection to compensate the sideslip angle does not decrease with $M_\infty$, as depicted in Fig. 14a. The reason for this trend is related to two main effects. First, at fixed $\alpha$ and $\beta$, the forces and moments acting on the aircraft increase with $M_\infty$. However, this effect is largely compensated by the lower required $\beta$ with increasing $M_\infty$ (see Table 1). The second effect is related to the difference in aerodynamic behavior below and above $M_\infty = 0.6$, resulting from transonic effects. In fully subsonic conditions and with fixed $\beta$, $C_\text {n}$ decreases with $\alpha$. Conversely, in transonic conditions, yawing moment coefficient increases with $\alpha$. For this reason, in transonic conditions, the required deflections increase (Fig. 14a). It should also be noted that the control authority of the rudder decreases with $\alpha$, which explains the large peak in required rudder deflection at $M_\infty =0.25$.

Figure 14b depicts the rudder deflections for the one-engine-inoperative requirement, showing for subsonic cases higher rudder deflections than for the sideslip cases with the exception of $M_\infty$ = 0.4. Differently, the rudder deflections are lower for the one-engine-inoperative requirement than sideslip cases at higher $M_\infty$. Once again, the requirement at $M_\infty$ = 0.2 is the highest and surpasses the limits considered in Table 4.

The rudder requirement for the one-engine-inoperative condition at $M_\infty$ = 0.2 of $\delta _r \simeq$ 40$^\circ$ is considered too large. The rudder requirement could be reduced by employing an additional control surface as the drag rudder. Here, the drag rudder is not sized as the other control surfaces, but the dimension and deflection required to unload the rudder are estimated based on [20]. The drag rudder is located on the outer part of the elevon, as visible in Fig. 9. It comprises two surfaces that move as one and in combination with the rest of the elevon, but the two surfaces can open, inducing drag and hence a yawing moment when required at low speed. On military airplanes, a combination of an aileron and drag rudder is sometimes referred to as a "splitteron". In the current implementation, this surface acts as a drag rudder, elevator, and aileron at the same time. The drag rudder has a span of 0.05*b*. Supposing that the maximum rudder deflection is $\delta _\text {r}$ = 30$^\circ$, the drag rudder should be deflected at ca. 30$^\circ$ to compensate the one-engine-inoperative yawing moment; at sea level and $M_\infty =0.2$ with a flap deflection of $\delta _\text {f}=40^\circ$, which leads to $\alpha$ = 10.5$^\circ$. Here, it is supposed that the effects of the drag rudder and of the rudder are superposable; however, this hypothesis will be tested in future studies.

When combining the sideslip and the one-engine-inoperative requirements, the maximum rudder deflections needed are occurring at $M_\infty$ = 0.2 and 0.25 amounting to ca. 32$^\circ$, supposing the use of the drag rudder at $M_\infty$ = 0.2.

Overall, these results show a different trend of the rudder deflection with Mach number compared to those for the elevon/elevator and the aileron. For elevon/elevator and aileron, the requirements in terms of deflection angles tend to decrease with $M_\infty$. Conversely, the requirement for the rudder increases with $M_\infty$ to satisfy the sideslip requirement, while showing a similar trend to elevon/elevator and aileron for the one-engine-inoperative condition.

### Coordinated turn

**Fig. 15:**
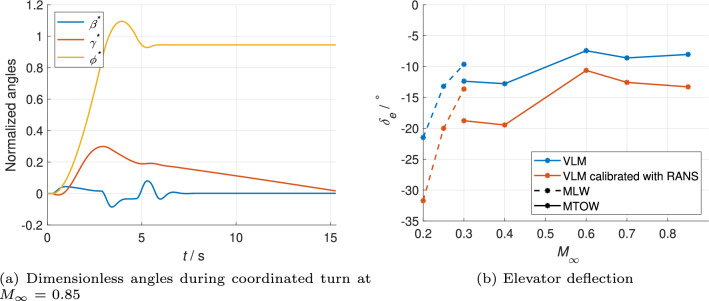
Angles evolution for coordinated turn and elevator deflection for coordinated turn at various flight conditions and at $x_{\text {CG}}$ = forw$_0$

Based on the results shown in Section 2.1.1, the coordinated turn is utilized as a verification maneuver to assess the final sizing of the control surfaces. As mentioned in Section 2.1.4, executing this maneuver requires reaching and maintaining a bank angle $\phi$ between 40$^\circ$ and 45$^\circ$ while keeping the sideslip angle $\beta$ as close to zero as possible, with a flight path angle $\gamma$ between 0$^\circ$ and 5$^\circ$. The maneuver is considered successful if these margins are satisfied for 10 s. Figure 15a shows the time history of the required angles. The angles are nondimensionalized for ease of comparison, where $\beta ^* = \beta / (max(\beta ) - min(\beta ))$, $\gamma ^* = \gamma / max(\gamma )$ and $\phi ^* = \phi / max(\phi )$. The max and min are the indicated margins above.

Figure 15a shows, for $M_\infty$ = 0.85, that with the layout of the selected control surfaces, it is possible to perform a coordinated turn within the limits defined by the certification specifications. Similar results are obtained in the other operating conditions considered in this study (Table 3). The most demanding requirements are for the elevon and elevator, which is shown in Fig. 15b. The required deflections are higher than those for the longitudinal trim in Section 3.3. In particular, as seen for all the cases so far, the highest $\delta _e$ is required at $M_\infty$ = 0.2, leading to $\delta _\text {e}$ = −32$^\circ$, which is higher than the deflections used in conventional aircraft.

### Discussion

The study so far conducted has focused on operating conditions, which should fall inside the flight envelope, helping to analyze how the requirements for control surfaces change when using an aerodynamic model, which includes nonlinearities, such as the pitch break.

However, the offline simulations also allows to determine speeds which are important to characterize the aircraft. The rotation speed, approach speed, and landing speed are a function of the stall speed. The stall speed is calculated here as the lowest speed at which the aircraft can be trimmed. Hence, the stall speed considered here is not directly comparable with the stall speed of a conventional aircraft, because the limit factor here is not the maximum lift coefficient but the occurrence of the pitch break [1].

The stall speed is calculated at the two CG locations discussed in Section 2.3. Additionally, for each CG position, two cases are tested, with and without high-lift flap, and finally, the calculations are conducted using MLW and MTOW. The stall speeds for the different cases are summarized in Table 8.

**Table 8:** Trimmed stall speed with and without high-lift at forwards and aft CG position

	$x_\text {CG}$	$\delta _\text {f}$	$\delta _\text {e}$	$U_\text {stall}$/(m/s)	$U_\text {stall}$/(knots)	$M_\text {stall}$
MLW	forw$_1$	0$^\circ$	−9$^\circ$	71	139	0.21
MLW	aft	0$^\circ$	−4$^\circ$	75	146	0.22
MLW	forw$_1$	40$^\circ$	−8$^\circ$	68	132	0.2
MLW	aft	40$^\circ$	−3$^\circ$	71	139	0.21
MTOW	forw$_2$	0$^\circ$	−7$^\circ$	82	159	0.24
MTOW	aft	0$^\circ$	−4$^\circ$	82	159	0.24
MTOW	forw$_2$	20$^\circ$	−6$^\circ$	78	152	0.23
MTOW	aft	20$^\circ$	−3$^\circ$	78	152	0.23

Identifying the lowest speed at which the aircraft could be trimmed corresponds to finding the highest $C_L$ before the pitch break region. Table 8 shows that the stall speed increases with weight and decreases when the high-lift flap is employed, as expected. At MLW, moving from the most forward to the most aft CG location, the stall speed increases. This is counterintuitive, but can be explained by the interaction between the elevator deflection and the stall over the outboard wing. At forward CG, the elevator and elevon are deflected trailing-edge up, which postpones stall over the outer wing. Therefore, the pitch break occurs at a higher angle of attack, corresponding to a higher lift coefficient. At MTOW, the stall speed between the forward and aft CG locations is shown to be the same. This is because the difference in speed is lower than $M_\infty$ = 0.005, which is the interval in Mach number for which the Mach trim routines are performed. For both the MLW and MTOW cases, the elevator deflection decreases when the CG is moving aft, as is expected.

As seen during the control surface sizing, an increase in speed leads to a lower deflection required by the control surfaces. For this reason for a more conservative analysis, the two lowest speeds identified in Table 8 are used as stall speeds, $U_{stall}$ = 68 m/s at MLW and $U_{stall}$ = 78 m/s at MTOW, to calculate the related speeds

- At MLW, $U_\text {approach}$ = 1.23 $\cdot$ $U_\text {stall}$ = 84 m/s = 162 knots ($M_\text {approach}$ = 0.25)
- at MTOW, $U_\text {rotation}$ = 1.2 $\cdot$ $U_\text {stall}$ = 94 m/s = 182 knots ($M_\text {rotation}$ = 0.28).

At the identified rotation speed, the take-off rotation requirement leads to an additional elevator and elevon deflection of ca. −8$^\circ$, leading to $\delta _\text {e}> - 25^\circ$, when using the high-lift flap deflected at 20$^\circ$.

Regarding the pull-up and push-over maneuvers, the former is the critical one, because it requires a more negative elevator and elevon deflection than the trim condition. Particularly, to obtain a load factor of 1.3 at $U_\text {approach}$ in combination with the high-lift flap, the additional requirement leads to $\delta _\text {e}> -25^\circ$.

Table 9 summarizes for all tested requirements which is the most critical condition in terms of speed and weight with the control surfaces as sized in this paper (Fig. 9). For all cases, a high-lift flap is required, to obtain deflections smaller than 32$^\circ$. The highest deflections are always reached with the elevator and elevon used together as elevator. The rudder deflection is limited at 30$^\circ$ assuming that a drag rudder is added to increase the yawing moment further.

Finally, it should be noted that because the elevon is used at the same time as aileron and elevator, when two maneuvers are combined, such as during longitudinal trim and a bank-to-bank maneuver, the deflection of the elevon would exceed 40$^\circ$ which is unrealistic. Apart from the take-off rotation and the pull-up at approach speed cases, the results are shown at stall speed. However, the approach speed or landing speeds are always greater than the stall speed, and as seen throughout this work, an increase in speed greatly decreases the maximum deflection required as does the deployment of a high-lift flap at low speeds.

**Table 9:** Summary of various requirements at most critical conditions and at $x_{\text {CG}}$ = forw$_0$

Certification req.	Speed	Weight	Max $|\delta |$	Addition
Longitudinal trim	Stall speed	MLW	$\delta _\text {e}>$ - 25$^\circ$	High-lift $\delta _\text {f}$ = 40$^\circ$
Pull-up	Approach speed	MLW	$\delta _\text {e}>$ - 25$^\circ$	High-lift $\delta _\text {f}$ = 40$^\circ$
Coordinated turn	Stall speed	MLW	$\delta _\text {e}>$ - 32$^\circ$	High-lift $\delta _\text {f}$ = 40$^\circ$
Take-off rotation	Rotation speed	MTOW	$\delta _\text {e}>$ - 25$^\circ$	High-lift $\delta _\text {f}$ = 20$^\circ$
Bank-to-bank	Stall speed	MLW	- $20^\circ< \delta _\text {a} < 20^\circ$	High-lift $\delta _\text {f}$ = 40$^\circ$
Sideslip	Stall speed	MLW	- $25^\circ< \delta _\text {a} < 25^\circ$	High-lift $\delta _\text {f}$ = 40$^\circ$
OEI	Stall speed	MLW	- $30^\circ< \delta _\text {r} < 30^\circ$	High-lift $\delta _\text {f}$ = 40$^\circ$,
				Drag rudder $\delta _\text {dr}\simeq$ 30$^\circ$

## Conclusion

The present work proposes a control surface layout for a flying wing aircraft based on offline handling quality simulations. The simulations employ an aerodynamic model based on Reynolds-Averaged Navier–Stokes (RANS) and Vortex Lattice Method (VLM) simulations. The control authority of the elevator, aileron, and rudder is estimated both with VLM and VLM calibrated with RANS simulations.

Initial simulations show that the Dutch roll is stable for most flight conditions, resulting in Level 2 handling qualities based on MIL-STD-1797A. These results allow simulations to be performed that would excite the aircraft simultaneously longitudinally and laterally without causing divergent behavior. Several simulations are then conducted to size each control surface. In particular, longitudinal trim, pull-up and take-off rotation maneuver for the elevator, steady-heading sideslip and a one-engine-inoperative condition for the rudder, and steady-heading sideslip and bank-to-bank for the aileron. Finally, coordinated turns are performed to verify whether the sized control surfaces could also comply with this requirement.

The defined control surface layout comprises an elevator, an elevon and a rudder. Where the elevon acts simultaneously as elevator and aileron, having the same deflection rates of the aileron, which are ca. 40% higher than the rate of the elevator.

The control surfaces are sized to satisfy the certification requirements even at stall speed, which is always the most demanding case. However, at stall speed, a high-lift flap and a drag rudder are required to satisfy the certification requirements. Overall, the simulations showed that the certification requirements can be satisfied at stall speed with elevator and elevon deflections $\delta _\text {e}>$ - 32$^\circ$, elevon deflection $-25^\circ<\delta _\text {a} <$ 25$^\circ$, and rudder deflection $-30^\circ< \delta _\text {r} <$ 30$^\circ$. In a more realistic scenario, when the aircraft flies faster than the stall speed, the required deflections of the control surfaces are reduced and get closer to the maximum deflections of the control surfaces of conventional aircraft.

The control authority of control surfaces is estimated with VLM and VLM calibrated with RANS, showing that without RANS calibration, VLM would underestimate the deflections needed to satisfy the certification requirements up to 43%. This difference is mainly driven by the inability of VLM to adequately predict the reduction in control effectiveness with angle of attack.

## Data Availability

No datasets were generated or analyzed during the current study.

## List of symbols

$A_x$, $A_y$, $A_z$: acceleration along X, Y, Z axis, m/s2
*b*: wing span, m
$\overline{c}$: mean aerodynamic chord, m
*CG*: center of gravity, m
$C_{X}$, $C_{Y}$, $C_{Z}$: force coefficient along *x*, *y*, *z*
$C_{l}$, $C_{m}$, $C_{n}$: moment coefficient around *X*, *Y*, *Z*
$C_{l,\delta _a}$: rolling moment coef. due to $\delta _a$
$C_{m,\delta _e}$: pitching moment coef. due to $\delta _e$
$C_{n,\delta _r}$: yawing moment coef. due to $\delta _r$
*g*: gravitational acceleration, m/s2
*h*: altitude, m
*H*: transfer function
*I*: moment of inertia, kg$\cdot$m2
*J*: cost function
*K*: gain
*m*: aircraft mass, kg
*n*: number of peaks
$M_{\infty }$: freestream Mach number
*p*, *q*, *r*: angular velocity around *x*, *y*, *z*, rad/s
$p_{t,\infty }$: freestream total pressure, Pa
$$\hat{p}$$, $$\hat{q}$$, $$\hat{r}$$: dimensionless *p*, *q* *r*
$\dot{p}$, $\dot{q}$, $\dot{r}$: angular acceleration around X, Y, Z rad/s$^2$
$Re_{\overline{c}}$: mean chord Reynolds number
*s*: Laplace variable (for continuous-time systems)
*t*: time, s
*T*: thrust, N
$T_{t,\infty }$: freestream total temperature, K
$$U_{approach}$$: approach velocity, m/s
$U_{des}$: desired freestream velocity, m/s
$$U_{\infty }$$: freestream velocity, m/s
$$U_{rotation}$$: rotation velocity, m/s
$$U_{stall}$$: stall velocity, m/s
$U_{trim}$: freestream trim velocity, m/s
*x*: axis and coordinate along chord direction, m
*y*: axis and coordinate along span direction, m
*z*: axis and coordinate along vertical direction, m
x$$_{hinge}$$: hinge line
$\alpha$: angle of attack, $^\circ$
$$\beta$$: sideslip angle, $$^\circ$$
$\delta _a$, $\delta _e$, $\delta _r$: aileron, elevator, rudder deflection angle, $^\circ$
$\delta _f$: high-lift flap deflection angle, $^\circ$
$\dot{\delta }$: control surface deflection rate, $^\circ$/s
$\omega _n$: natural frequency, rad/s
$\rho _{\infty }$: freestream density, kg/m$^3$
$$\phi$$: bank angle, $$^\circ$$
$$\theta$$: pitch angle, $$^\circ$$
$$\zeta$$: damping ratio
$\omega$: natural frequency, rad/s
aft: aft CG location
act: actuator
eng: engine
forw: forward CG location
BPR: Bypass ratio
CG: Center of Gravity
CV: VLM calibrated with experiment
HQ: handling quality
MTOW: Maximum take-off weight, N
MLW: Maximum landing weight, N
OEW: Operative empty weight, N
RANS, R: Reynolds-averaged Navier-Stokes
SLS: Sea-level static
SQP: Sequential quadratic programming
VLM: Vortex Lattice Method
